# Laser Tracker and Terrestrial Laser Scanner Range Error Evaluation by Stitching

**DOI:** 10.3390/s24102960

**Published:** 2024-05-07

**Authors:** Bala Muralikrishnan, Braden Czapla, Vincent Lee, Craig Shakarji, Daniel Sawyer, Matthias Saure

**Affiliations:** 1 Sensor Science Division, National Institute of Standards and Technology, Gaithersburg, MD 20899, USA; 2 Leica Geosystems AG, CH-5035 Unterentfelden, Switzerland

**Keywords:** laser tracker, overlap, range error, registration, stitching errors, stitching lengths, terrestrial laser scanner, uncertainty

## Abstract

Laser trackers (LTs) are dimensional measurement instruments commonly employed in the manufacture and assembly of large structures. Terrestrial laser scanners (TLSs) are a related class of dimensional measurement instruments more commonly employed in surveying, reverse engineering, and forensics. Commercially available LTs typically have measurement ranges of up to 80 m. The measurement ranges of TLSs vary from about 50 m to several hundred meters, with some extending as far as several kilometers. It is difficult, if not impossible, to construct long reference lengths to evaluate the ranging performances of these instruments over that distance. In this context, we explore the use of stitching errors (i.e., stacking errors in adjoining or overlapping short lengths) and stitching lengths (i.e., constructing long reference lengths from multiple positions of a reference instrument by registration) to evaluate these instruments. Through experimental data and a discussion on uncertainty, we show that stitching is indeed a viable option to evaluate the ranging performances of LTs and TLSs.

## 1. Introduction

Laser trackers (LTs) [1] are portable large-scale dimensional measurement instruments that measure the three-dimensional coordinate of a cooperative target (such as a spherically mounted retroreflector (SMR)) by recording the range, the azimuth angle, and the elevation angle to the target. They are typically used in the manufacture and assembly of large structures, the alignment of components, error mapping machine tools and robots, etc. Commercially available LTs have a maximum range of about 80 m when used in conjunction with an SMR 38.1 mm (1.5 in) in diameter. The ranging unit of the LT provides the connection to the SI unit of length, the meter. Verifying that the ranging unit performs within the manufacturer’s accuracy specifications is, therefore, important. 

Terrestrial laser scanners (TLSs) [2,3,4] are also portable large-scale dimensional measurement instruments that measure three-dimensional coordinates by recording the range, the azimuth angle, and the elevation angle of points in the scene. TLSs, however, do not require a cooperative target. They are used in geodesy and surveying, reverse engineering, forensic investigations, historical monument preservation, etc. Several commercially available TLSs have maximum ranges of several hundred meters, while some can measure up to several kilometers. As in the case of LTs, verifying the ranging performances of TLSs is important because the ranging unit provides the connection to the SI unit of length, the meter. Note that the ranging evaluation of LTs and TLSs is only one part of the overall testing process, which typically involves volumetric testing (for angular errors), level sensor performance testing, etc.

There are two significant challenges in verifying the ranging performance of LTs and TLSs. The first concerns the accuracy of the reference instrument used in the evaluation process and whether it is small enough compared to the accuracy specification of the ranging unit of the LT or TLS under test. The second challenge is a practical limitation faced by most users and even manufacturers of LTs and TLSs: constructing and maintaining an environmentally controlled room large enough to test the full range of an LT or a TLS can be prohibitively expensive. Even though TLSs are often used in outdoor environments, testing them in a controlled environment is important to assess the performance of the ranging unit without the environmental effects (and errors in the TLS’s environmental sensors, if any) influencing the test.

In the case of LT range verification, a laser interferometer is commonly used as the reference instrument. Its frequency can be calibrated, and its range is comparable to that of an LT. However, its accuracy is similar to the accuracy of the interferometric ranging unit of an LT under test; thus, it is not possible to achieve the 4:1 (or better) accuracy ratio desired in a testing process (see ASME B89.7.3.1-2001 [5] for more on this). With regard to the practical limitation of room sizes, the tape tunnel facility [6] at the National Institute of Standards and Technology (NIST), which is the National Metrology Institute (NMI) of the United States, has an interferometric bench that is only 60 m long. While we can test the full 80 m range of LTs using only 40 m of the bench through the double-pass method [7], most users or even non-NMI laboratories will likely not have a bench that is 40 m long. 

In the case of TLS range error verification, a total station is often used as the reference instrument, but its accuracy is not substantially smaller than the ranging accuracy specifications of a TLS. An LT offers high accuracy but is limited in its range. With regard to the practical limitation of room size, it would be extraordinarily challenging to construct an environmentally controlled chamber hundreds of meters long to evaluate the ranging unit of a TLS. However, the ranging unit could be evaluated in long corridors in buildings where temperature is moderately controlled or outdoors where environmental effects are likely the largest contributor to ranging errors of the TLS. 

We propose stitching as a solution to some of the challenges listed above. We consider two approaches to stitching. In the first case, we stitch errors of adjoining or overlapping short lengths to evaluate the errors of a long length. Note that we do not actually construct long reference lengths; instead, we directly estimate the errors of those lengths. In the second case, we construct long reference lengths by transforming reference instrument data from different positions to a common frame by registration. That is, we stitch short reference lengths to construct long reference lengths and use that to calculate the errors of the instrument under test. 

In the case of LTs, the stitching of errors allows users to construct a testing facility that is substantially smaller than the full range of an LT, thus addressing the second challenge of room size. Stitching lengths are not possible for LTs when an interferometer is used as the reference instrument because it does not provide the 3D coordinates required for registration. In the case of TLSs, the stitching process (both errors and lengths) using an LT as the reference instrument allows users to achieve uncertainties in the reference lengths that are substantially smaller than the ranging accuracy specifications of TLSs, thus addressing the first challenge of sufficient reference instrument accuracy. 

We demonstrate the stitching process for LTs by comparing the ranging errors of the interferometer (IFM) of an LT over a single 60 m length and the ranging errors obtained by stitching errors in overlapping 30 m lengths using the common path single-pass method (see Section 4.1). We also demonstrate the stitching process by comparing the ranging errors of the IFM over a single 76 m length (ADM signal deteriorated at about 78 m in this setup, so we could not measure up to 80 m) and the ranging errors obtained by stitching errors in overlapping 38 m lengths using the common path double-pass method (see Section 4.2). We perform similar comparisons for the absolute distance meter (ADM) of the LT.

We demonstrate the stitching process for TLSs in our temperature-controlled tape tunnel facility by comparing the ranging errors over a single 60 m length, ranging errors obtained by stitching errors in overlapping 30 m lengths (see Section 5.1), and ranging errors obtained by stitching reference lengths to construct long reference lengths. We also demonstrate the stitching process in a long, moderately temperature-controlled indoor corridor by comparing the ranging errors over 120 m obtained by stitching errors in overlapping 60 m lengths (see Section 5.2) and the ranging errors obtained by stitching reference lengths. 

The idea of stitching itself is not new (as we show in the literature review in the next Section). The main contribution here is the application of stitching to characterize ranging errors of LTs and TLSs over long distances (>10 m). The rest of this paper is organized as follows. We present a review of the literature in Section 2, a general discussion on stitching in Section 3, the application of stitching to LTs and TLSs in Section 4 and Section 5, respectively, a short discussion on test value uncertainty in Section 6, and conclusions in Section 7.

## 2. Literature Review

### 2.1. LT Range Evaluation

Test procedures to evaluate the ranging performance of an LT are described in both the ASME B89.4.19-2021 [8] and ISO 10360-10-2021 [9] standards. For example, the ASME B89.4.19 recommends the evaluation of the relative range errors at four positions for the IFM and six positions for the ADM of an LT. The standard does recognize that it may be difficult to obtain expanded uncertainties (*k* = 2) in the reference values that are smaller by a factor of at least 4 when compared to the manufacturer’s specification for ranging errors, and thus, a factor of 2 is allowed. The practical realization of ranging tests using a long interferometric bench is realized by NMIs around the world, as shown in Table 1. 

In addition, the Physikalisch-Technische Bundesanstalt (PTB), Germany, [16], the National Physical Laboratory (NPL), UK, [17], and the Korea Research Institute of Standards and Science (KRISS) [18] also have 50 m long interferometric benches. 

Academic institutions and other non-NMI laboratories have also established interferometric bench setups for LT range error characterization. For example, the Czech Technical University in Prague [19] and the Stanford National Accelerator Laboratory [20] maintain 30 m benches for LT range error evaluation. The European Synchrotron Radiation Facility in France [21] has a 50 m bench, while the Trescal calibration facility [22] has a 21 m bench. 

### 2.2. TLS Range Evaluation

TLS range errors are generally evaluated against calibrated baselines realized using stable pillars spread over hundreds of meters. An in-depth review of ranging errors for TLS measurements is provided by Muralikrishnan [4]. A brief summary of that work is presented here for completeness. Rüeger [23] provides a detailed overview of baseline designs for EDM calibrations. Early baseline pillar-based TLS measurements are described by Gordon et al. [24] and Kersten et al. [25]. While baseline pillars allow a range error evaluation over large distances, environmental effects play a significant role in the measurements, and therefore, it is difficult to evaluate the intrinsic ranging error of the instrument under test. Ranging tests have also been reported in more controlled environments against reference instruments of higher accuracy. For example, Boehler et al. [26] used an interferometer to evaluate a TLS over a short range (<10 m). Ingensand et al. [27] reported on a range of measurements on a TLS performed on a 52 m calibration track, where the reference values were established using an interferometer. Johansson et al. [28], Mechelke et al. [29], Gonzalez-Jorge et al. [30], and Lee et al. [31] describe the ranging tests of TLS systems by comparing them against a total station. Salo et al. [32] and Staiger and Ettel [33] use a tacheometer as a reference to evaluate TLS range errors. Fuss et al. [34] and Ferrucci et al. [35] use a laser tracker as a reference instrument for TLS range error evaluation. 

Standardized test procedures to evaluate the ranging performance of a TLS are available in the ASTM E2938-15 [36] and ASTM E3125-17 [37] standards. These standards describe the use of a plate target that is measured by both the TLS and a reference instrument in order to evaluate the ranging errors. 

### 2.3. Stitching (with or without Fold Mirrors)

As we noted in Section 1, one of the main challenges with range error evaluation of LTs is the physical size of the facility. Turolski and Turolski [38] describe a 30 m long interferometric bench constructed in 10 m segments using mirrors to fold the beam path. They report on measurements of a 3 m length placed at different positions in the path to estimate errors as a function of distance from the LT. Linville [7] reports on a 27 m interferometric bench used to evaluate an LT for twice that distance using a clever arrangement of mirrors and SMRs. Khalil [39] describes the use of mirrors to create a long reference length so that a TLS may be evaluated inside a building of limited size. These works do use a mirror to fold the beam to save space, but they do not construct a long reference length by stitching.

In the case of Cartesian coordinate measuring machines (CMMs), the idea of stitching short segments to construct a longer length has been considered by Cox et al. [40] and Icasio-Hernandez et al. [41] and allowed in Annex-B of ASME B89.4.10360-2 [42]. Cox et al. considered the problem of calibrating an artifact that is longer than the range of a CMM. and Icasio-Hernandez et al. considered this problem as well as the inverse problem, i.e., using a short artifact to verify the performance of a CMM of longer range. This second problem is really the topic of concern here. In the case of laser trackers, Lee et al. [43] considered the problem of stitching a 1.5 m reference length to construct an effective 3 m reference length for evaluating the angular errors of an LT. Shi et al. [44] performed the same for the case of TLSs. But these works describe the use of stitching to create a reference length that is still short at under 10 m. 

To the best of our knowledge, there is no reported research in the literature that considers stitching short reference lengths to create a long reference (i.e., several tens to hundreds of meters), with or without the use of fold mirrors, for evaluating the ranging errors of LTs and TLSs. Stitching is, however, allowed in the ISO 10360-10 standard [9] for LTs, but no guidance is provided on how to realize this in practice. We, therefore, address the issue of stitching in this paper. 

## 3. Stitching

We describe two ways of stitching in this paper—stitching errors and stitching lengths. In the case of stitching errors, we stack errors in adjoining or overlapping short lengths to compute the overall ranging error of a long length. In the case of stitching lengths, we use stationary registration nests to transform data from different positions of the reference instrument to a common coordinate system, so that we can obtain reference distance values and, therefore, the ranging errors over the full testing range of the instrument under test. We describe these methods in more detail next.

### 3.1. Stitching Errors

**Figure 1:**
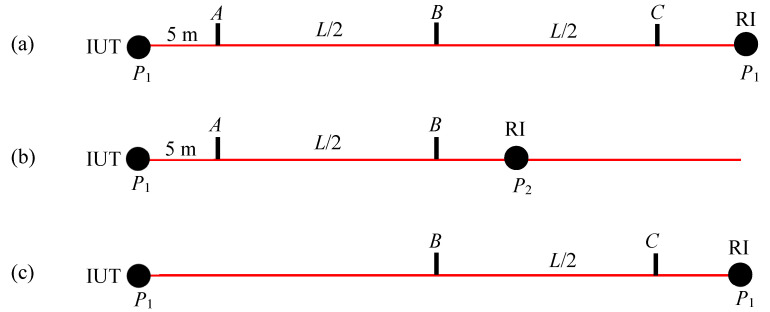
Basic idea of stitching errors for range error evaluation. (**a**) Measuring the errors at target positions *B* and *C* with respect to position *A* in one setup if the RI has the same range as the IUT, (**b**) measuring the error between target positions *A* and *B* with the RI at *P*_2_, and (**c**) measuring the error between target positions *B* and *C* with the RI at *P*_1_.

Suppose we are interested in evaluating the ranging performances of an instrument under test (IUT). Consider three positions, *A*, *B*, and *C*, over the testing distance *L,* as shown in Figure 1. We consider position *A* as the reference position for purposes of range error evaluation, i.e., range errors are calculated at different positions (e.g., *B* and *C*) with respect to *A*. The reference position, if not specified by the manufacturer, is arbitrarily chosen. If we have access to a long room and a means of establishing the reference values over the full length of interest (*L* m in Figure 1a), we can perform this measurement in a single setup, i.e., by measuring the positions of a target at *A*, *B*, and *C*, sequentially using both the IUT and the reference instrument (RI), and then calculate the errors at *B* and *C* with respect to *A*.

Suppose we are limited by a reference instrument that only has half the range of the IUT, such as might be encountered for TLS testing. Then, we can first measure the distance between a target at positions *A* and *B* (Figure 1b), and then, for the second measurement, the distance between a target at positions *B* and *C* (Figure 1c). Note that target position *B* (with respect to the IUT) does not have to be coincident for the two measurements. Because the ranging errors are systematic and slowly vary with range, and we are not calculating the length *AC*, position *B* can be within about 0.1 m or so between the two measurements. Let the error in the segments *AB* and *BC* be *e_AB_* and *e_BC_*, respectively. Then, the error in the overall length *AC*, *E_AC_*, can be obtained by stitching, i.e., as the sum of the errors of the individual segments *AB* and *BC*. Thus, *E_AC_* = *e_AB_* + *e_BC_*. We discuss the implications of stitching errors on the test value uncertainty (i.e., uncertainty in the errors) in Section 6. 

**Figure 2:**
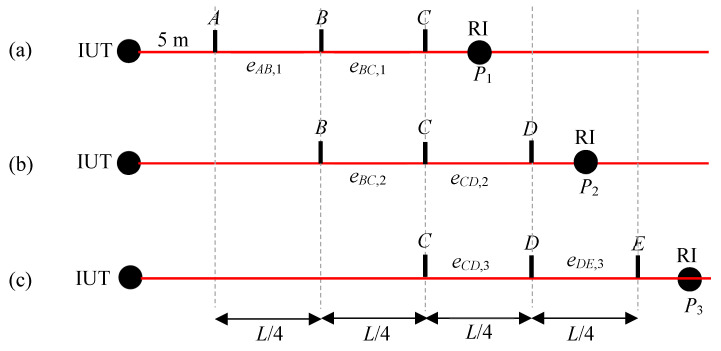
Stitching error from overlapping segments. (**a**) Measuring the target at positions *A*, *B*, and *C* with the RI at *P*_1_, (**b**) measuring the target at positions *B*, *C*, and *D* with the RI at *P*_2_, and (**c**) measuring the target at positions *C*, *D*, and *E* with the RI at *P*_3_.

To reduce the influence of the random errors, we can average multiple measurements of the IUT at each of the positions A, B, and C. An alternate method of averaging not only reduces the effect of random errors but also increases the resolution of the range error map by introducing additional sampling points. The approach is shown in Figure 2, where we consider four segments, each of length *L*/4, over the range *L* of the IUT, and three positions for the RI. We first position the RI at *P*_1_ and measure the target with both the RI and IUT at positions *A*, *B*, and *C*. We calculate the errors *e_AB_*_,1_ and *e_BC_*_,1_. These are the errors of the individual segments *AB* and *BC* with the RI at *P*_1_. Note that the second subscript (1) for the error *e* refers to the RI position. We can then move the RI to *P*_2_, measure the target with both the RI and IUT at positions *B*, *C*, and *D*, and calculate the errors in the individual segments. Finally, we move the RI to *P*_3_, measure the target with both the RI and IUT at positions *C*, *D*, and *E*, and calculate the errors in the individual segments. 

We calculate the final ranging error at positions *B*–*E* as follows. The ranging error is zero at position *A* because it is the reference for ranging errors. Because segment *AB* is only measured from position *P*_1_, the ranging error at *B* (with respect to *A*) is simply *e_AB_*_,1_. Because there are two measurements of segment *BC* (with the RI at *P*_1_ and *P*_2_), we can consider the average as the estimate of the error for that segment, i.e., *e_BC_* = (*e_BC_*_,1_ + *e_BC_*_,2_)/2. The overall ranging error at *C* (i.e., with respect to *A*) is the sum of the errors of segments *AB* and *BC*; thus, *E_C_* = *e_AB_*_,1_ + (*e_BC_*_,1_ + *e_BC_*_,2_)/2. Following this logic, the ranging errors at the different target positions are as follows:

*E_A_* = 0 (1)

*E_B_* = *e_AB_*_,1_

*E_C_* = *e_AB_*_,1_ + (*e_BC_*_,1_ + *e_BC_*_,2_)/2

*E_D_* = *e_AB_*_,1_ + (*e_BC_*_,1_ + *e_BC_*_,2_)/2 + (*e_CD_*_,2_ + *e_CD_*_,3_)/2

*E_E_* = *e_AB_*_,1_ + (*e_BC_*_,1_ + *e_BC_*_,2_)/2 + (*e_CD_*_,2_ + *e_CD_*_,3_)/2 + *e_DE_*_,3_

We can increase the number of segments and the number of RI positions to reduce the influence of random noise and produce a higher-resolution map of the ranging errors. But doing so also increases the amount of time and effort it takes to perform the measurement. 

### 3.2. Stitching Lengths

**Figure 3:**
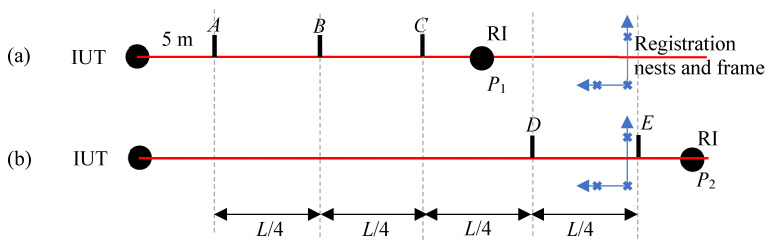
Stitching lengths by registration, (**a**) measuring the target at positions *A*, *B,* and *C*, and the registration nests with the RI at *P*_1_ and (**b**) measuring the target at positions *D* and *E*, and the registration nests with the RI at *P*_2_.

Instead of stitching errors, as we have described in the previous section, we can construct a long reference length using stationary registration nests to bring the RI data to a common coordinate system if the RI can produce 3D point coordinates. Consider the RI (whose range is smaller than that of the IUT) located at position *P*_1_, as shown in Figure 3a. We record the RI and IUT target center coordinates at positions *A*, *B*, and *C*. We also measure the three registration nests using the RI. While the data acquired by the IUT is always in the IUT coordinate system, we transform the RI data to a coordinate system defined by the registration nests; thus, the RI coordinates for *A*, *B*, and *C* are in the registration frame. We then move the RI to position *P*_2_, as shown in Figure 3b. We record the RI and IUT target center coordinates at positions *D* and *E*. We measure the three registration nests using the RI, construct a new registration frame, and transform the RI data into the registration frame. As a result, the RI target centers for all positions *A*–*E* are obtained in one coordinate system. We can, therefore, calculate the ranging errors at positions *B*–*E* with respect to *A*. We discuss the implications of stitching errors on the test value uncertainty (i.e., uncertainty in the errors) in Section 6.

**Figure 4:**
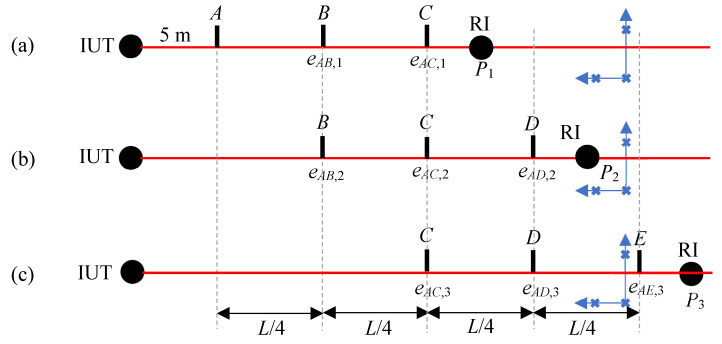
Stitching lengths by registration. (**a**) Measuring the target at positions *A*, *B,* and *C*, and the registration nests with the RI at *P*_1_, (**b**) measuring the target at positions *B*, *C*, and *D*, and the registration nests with the RI at *P*_2_, and (**c**) measuring the target at positions *C*, *D*, and *E*, and the registration nests with the RI at *P*_3_.

Increasing the number of nests used in the registration can help attenuate this problem to some extent. Overlapping segments also help to reduce the influence of random errors. In Figure 4, we consider four segments, each of length *L*/4, over the range *L* of the IUT, and three positions for the RI. As we move the RI to different positions, we measure the registration nests from each position. Targets at some positions are, therefore, measured from multiple RI positions. As in the case of stitching errors, because the ranging errors are systematic and slowly vary with range, and we are always calculating the error with respect to position A, the target positions at any given location (for example, position C with the RI at *P*_1_, *P*_2_, and *P*_3_) can be within about 0.1 m or so between the measurements.

From the measured data, we can calculate a ranging error at target position *B* from both RI positions *P*_1_ and *P*_2_. We can calculate a ranging error at target position *C* from all three RI positions. We can calculate a ranging error at target position *D* from two RI positions, *P*_2_ and *P*_3_. At each target position, we can average the errors from the different RI positions to reduce the influence of random errors. Note that the errors are directly obtained with respect to target position *A*. They are, therefore, shown at the target position in Figure 4 instead of between target positions as in Figure 2. This is also seen in the first subscript for the error *e*, which is always *A*. The ranging errors at target positions *A*–*E* are as follows:

*E_A_* = 0 (2)

*E_B_* = (*e_AB_*_,1_ + *e_AB_*_,2_)/2

*E_C_* = (*e_AC_*_,1_ + *e_AC_*_,2_ + *e_AC_*_,3_)/3

*E_D_* = (*e_AD_*_,2_ + *e_AD_*_,3_)/2

*E_E_ = e*_*AE*,3_

## 4. LT Range Error Evaluation

We use an interferometer as the reference instrument for LT range error evaluation. Because an interferometer does not provide 3D coordinates, we cannot perform LT range error evaluation by stitching lengths; thus, we could only show LT range error evaluation by stitching errors. We demonstrate the feasibility of stitching errors through two experiments in our tape tunnel facility. The facility has a reference interferometer and 60 m long rail and carriage assembly and is temperature-controlled to ±0.5 °C over the 60 m distance. The first experiment uses the common path single-pass technique, while the second experiment uses the common path double-pass technique [45]. The common path single-pass technique is easier to realize but we were limited by the length of our rail; thus, we could only test an LT up to 60 m. The common path double-pass technique is comparatively more involved, but we could test the full 80 m range of an LT using only 40 m of the bench. We performed stitching experiments for both the IFM and the ADM of the LT under test. In the next sub-sections, we describe the application of stitching using mirrors, summarize the single-pass and double-pass techniques for completeness, and present the results and a discussion on test uncertainty and MPE.

### 4.1. Stitching

**Figure 5:**
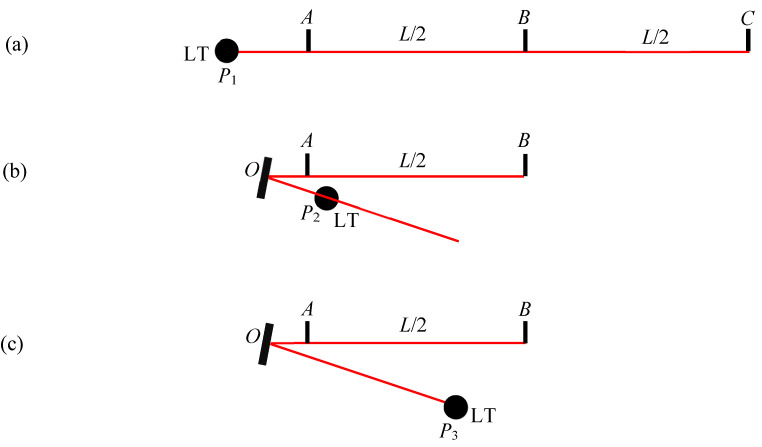
Basic idea of the stitching operation for LT range error evaluation. (**a**) Measuring the target at positions *A*, *B*, and *C* with the LT at *P*_1_ (reference interferometer not shown), (**b**) measuring the target at positions *A* and *B* with the LT at *P*_2_ and using a mirror close to position *A*, (**c**) measuring the target at positions *A* and *B,* with the LT at *P*_3_ (equivalent to measuring segment *BC* with LT at *P*_1_) and using the same mirror as before.

An interferometer and an LT typically have similar ranges. Thus, the range error measurement of an LT is not limited by the range of the reference instrument. Figure 5a shows the evaluation of an LT at position *P*_1_ when we had access to a room long enough to cover the full range (the reference interferometer is not shown). In that case, we could sequentially measure a target at positions *A*, *B*, and *C* and calculate the ranging errors at *B* and *C* with respect to *A*. However, most users of LTs do not have access to such a room. In that case, measurement by stitching can be performed as follows. We place the LT at position *P*_2_ and a mirror close to target position *A,* as shown in Figure 5b. We bounce the LT laser off the mirror so that the laser beam of the LT is in line with positions *A* and *B*. We can then measure the error *e_AB_* in segment *AB*. We then move the LT to position *P*_3_ and measure the error *e_BC_* in segment *AB*. Note that moving the LT to *P*_3_ is equivalent to measuring segment *BC* with the LT at *P*_1_ in Figure 5a. The error in the overall length *AC*, *E_AC_*, can be obtained by stitching, i.e., as the sum of the errors of the individual segments *AB* and *BC*. Thus, *E_AC_* = *e_AB_* + *e_BC_*. Note that the reference instrument is not shown in Figure 5. As mentioned in Section 3.1, we can increase the number of segments and the number of LT positions to reduce the influence of random noise and to produce a higher-resolution map of the ranging errors, but this takes more time and effort. 

In the case of LTs, the use of a mirror is only possible because the size of the laser spot is small, so a high-quality mirror of say, 25.4 mm (1 in) in diameter, may be used, and these mirrors are not very expensive. This advantage is realized when the mirror is closer to position *A,* as shown in Figure 5c. If the mirror is farther away from position *A*, the LT has to be closer to the mirror, requiring a large room to perform the measurement. Positioning the mirror close to one end of the length is important to realize the objective of being able to use a smaller room for the test.

### 4.2. Common Path Single-Pass

**Figure 6:**
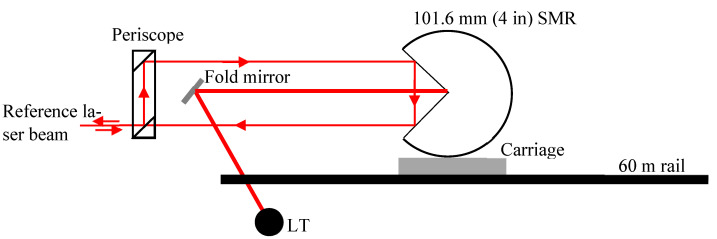
Common path single-pass method.

In the common path single-pass setup, the reference interferometer is placed at one end of the rail; see Figure 6. A moving carriage holds a very large 101.6 mm (4 in) SMR and faces the reference laser. The outgoing beam from the reference laser is bent using a periscope so that the beam strikes the SMR away from its apex, bounces off the mirrors inside the SMR, and returns so that it is separated by about 25.4 mm (1 in) in the vertical plane. This separation allows for a small fold mirror to be mounted on a goniometer assembly. The laser beam from the LT bounces off the mirror, strikes the apex of the SMR, and returns to the LT via the mirror. This ‘common path’ approach allows the LT to be placed near the reference interferometer, and a single SMR is sufficient. The advantage of the common path setup is that the uncertainty of the reference and test measurements grows larger together. While we use a 101.6 mm (4 in) SMR as the reflector on the carriage, a cube corner reflector is sufficient for this purpose. See Blackburn et al. [10] for more on the common path setup. The common path setup is also described by Gruza et al. [46]. Haitjema [47] presents a general discussion of different setups for displacement interferometer evaluation.

**Figure 7:**
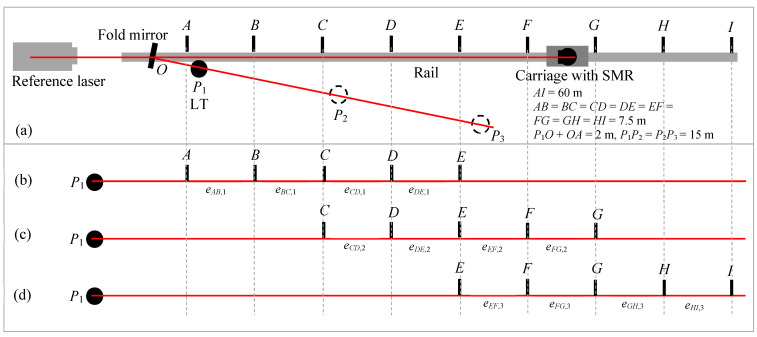
Common path single-pass approach. (**a**) Experimental setup showing the LT and carriage positions; (**b**) step 1 of the stitching process, measuring the errors at positions *A*–*E* with the LT at *P*_1_; (**c**) step 2 measuring the errors at positions *C*–*G* with respect to the LT, realized in practice by measuring positions *A*–*E* with the LT at *P*_2_; and (**d**) step 3 measuring the errors at positions *E*–*I* with respect to the LT, realized in practice by measuring positions *A*–*E* with the LT at *P*_3_.

The stitching experiment is performed as follows. We consider three positions for the LT (*P*_1_–*P*_3_) and nine positions for the carriage (*A*–*I*), as shown in Figure 7a. LT positions *P*_1_–*P*_3_ are nominally 15 m apart, and carriage positions *A*–*I* are nominally 7.5 m apart. Thus, the distance from *A* to *I* is 60 m. Position *P*_1_ is about 2 m from *A* (i.e., *P*_1_*O* + *OA* = 2 m), while positions *P*_2_ and *P*_3_ are 17 m and 32 m from *A*, respectively. Our objective is to stitch range errors of 30 m lengths to construct the error over 60 m in length. 

We first perform the range error measurement over the full 60 m distance to establish a reference against which we can compare the results obtained by stitching. For this purpose, the LT is located at *P*_1_. We record the laser tracker coordinates and the reference interferometer readings sequentially at the nine carriage positions *A*–*I*. We then calculate the range error of positions *B*–*I* with respect to position *A*. 

The stitching measurements are performed in three steps, as follows. For the first step, we record the laser tracker coordinates and the reference interferometer readings at the five carriage positions *A*–*E* with the LT located at *P*_1_, as shown in Figure 7b. For the second step, we move the LT to *P*_2_ and perform the measurements at carriage positions *A*–*E*. This is effectively the same as measuring positions *C*–*G* (as shown in Figure 7c) from LT position *P*_1_. For the third step, we move the LT to *P*_3_ and perform the measurements at carriage positions *A*–*E*. This is equivalent to measuring positions *E*–*I* (as shown in Figure 7d) from the LT position *P*_1_. Moving the LT for steps 2 and 3 allows us to use a smaller space to perform the measurements, which is the main objective of stitching. In the common pass single-path method combined with stitching, we can test an LT up to a range of about 60 m using a room that is about 35 m long. 

The data are analyzed, as described in Section 3.1. While we use an interferometer rail and carriage setup to demonstrate the stitching concept here, the discussion also applies to the case where a reference length is realized using nests on free-standing structures, such as stands.

### 4.3. Common Path Double-Pass

**Figure 8:**
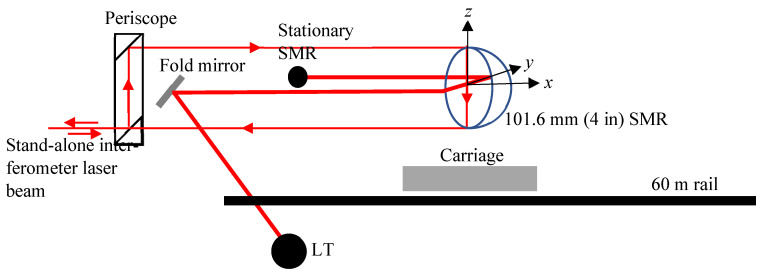
Common path double-pass test setup.

In order to measure the full range of the laser tracker, i.e., 80 m, we made a small modification to the common path single-pass method based on the work reported by Linville et al. [8]. We moved the fold mirror slightly to one side and installed a spherically mounted retroreflector (SMR) next to it so that the laser beam of the laser tracker bounced off the fold mirror, struck the 101.6 mm (4 in) SMR on one face of the cube corner (instead of at its apex), struck another face, and returned to lock onto the stationary SMR, as shown in Figure 8. The outgoing and incoming laser beams from the stand-alone interferometer are displaced in the vertical plane, while the outgoing and incoming laser beams of the laser tracker under test are displaced in the horizontal plane. When the carriage is moved by a certain amount, the laser tracker records twice the displacement seen by the reference. Thus, we can achieve an 80 m displacement for the laser tracker using only a 40 m displacement of the carriage. 

**Figure 9:**
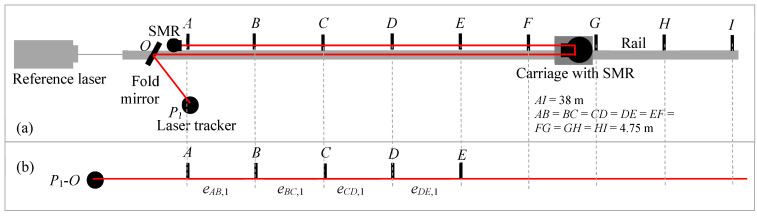
Common path double-pass approach. (**a**) Experimental setup showing the LT and carriage positions; (**b**) step 1 of the stitching process-measuring the errors at positions *A*–*E* with the LT at *P*_1_.

**Figure 10:**
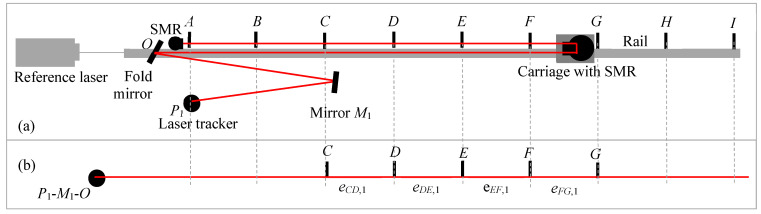
Common path double-pass approach. (**a**) Experimental setup showing the LT and carriage positions and mirror at *M*_1_; (**b**) step 2 measuring the errors at positions *C*–*G* with respect to the LT, realized in practice by measuring positions *A*–*E* with the LT at *P*_1_ and using a mirror at *M*_1_.

**Figure 11:**
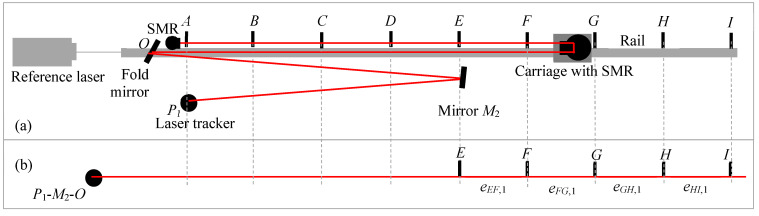
Common path double-pass approach. (**a**) Experimental setup showing the LT and carriage positions and mirror at *M*_2_; (**b**) step 3 measuring the errors at positions *E*–*I* with respect to the LT, realized in practice by measuring positions *A*–*E* with the LT at *P*_1_ and using a mirror at *M*_2_.

The stitching experiment is performed as follows. We consider nine positions for the carriage (*A*–*I*), spaced about 4.75 m apart, as shown in Figure 9a. Thus, the distance from *A* to *I* is 38 m. The LT is placed at position *P*_1_, which is about 2 m from *A* (i.e., *P*_1_*O* + *OA* = 2 m) for all measurements. We used two additional mirrors located at *M*_1_ and *M*_2_, as shown in Figure 10 and Figure 11. *M*_1_ was placed about 9.5 m from the LT, while *M*_2_ was about 19 m from the LT. Our objective was to stitch range errors in 38 m lengths to construct the errors over the 76 m length. The ADM signal deteriorated at 78 m (position *I* was 78 m from the LT, i.e., *P*_1_*O* + *OI* = 78 m); we, therefore, did not measure up to 80 m. 

We first performed the range error measurement over the full 76 m distance to establish a reference against which we could compare the results obtained by stitching. For this purpose, the LT was located at *P*_1_, and its laser bounced off mirror *O* to strike the SMR on the carriage and locked onto the stationary SMR located near *O*; see Figure 9a. We recorded the laser tracker coordinates and the reference interferometer readings sequentially at the nine carriage positions *A*–*I* with the LT located at *P*_1_. We then calculated the range error of positions *B*–*I* with respect to position *A*. 

The stitching measurements were performed in three steps, as follows. For the first step, we record the laser tracker coordinates and the reference interferometer readings at the five carriage positions *A*–*E* with the LT located at *P*_1_. We then calculated the errors in each segment, as shown in Figure 9b. 

For the second step, we aligned the mirror at *M*_1_ so that the laser beam of the LT reflected off this mirror, struck the mirror at *O*, then the SMR on the carriage, and finally locked on to the stationary SMR located near the mirror at *O*. We performed the measurements at carriage positions *A*–*E*. This is shown in Figure 10a. Bouncing the laser off the mirror at *M*_1_ and measuring target positions *A*–*E* was equivalent to measuring target positions *C*–*G* if the LT was in line with those positions, as shown in Figure 10b. We calculated the errors for each segment, as shown in Figure 10b. 

Finally, for the third step, we aligned the mirror at *M*_2_ so that the laser beam of the LT reflected off this mirror, struck mirror *O*, then the SMR on the carriage, and finally locked on to the stationary SMR located near the mirror at *O*. We performed the measurements at carriage positions *A*–*E*. This is shown in Figure 11a. Again, bouncing the laser off the mirror *M*_2_ and measuring target positions *A*–*E* was equivalent to measuring target positions *E*–*I* if the LT was in line with those positions, as shown in Figure 11b.

Bouncing the LT laser off the mirrors *M*_1_ and *M*_2_ in steps 2 and 3 allowed us to use a smaller space to perform the measurements, which was the main objective of stitching for the case of LTs. In order to test the 76 m range of the LT, we only required a room that was about one-fourth in length, i.e., about 20 m long. This is the significant advantage gained by stitching short reference lengths to construct the errors of the LT over its full range.

### 4.4. Results

**Figure 12:**
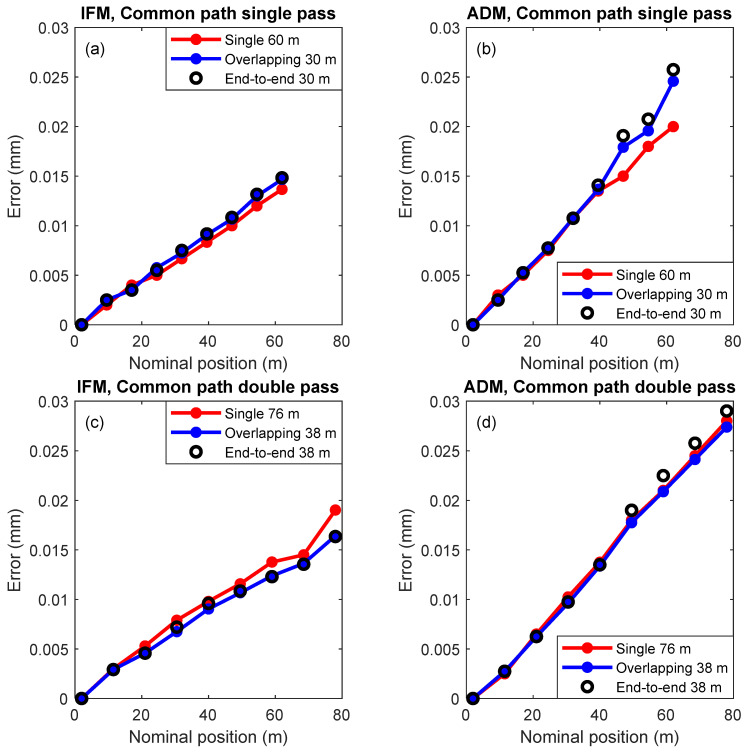
Common path single-pass results for the (**a**) IFM and (**b**) ADM. Common path double-pass results for the (**c**) IFM and (**d**) ADM. The figures show the ranging errors from a single measurement (red line), those obtained by stitching errors using three overlapping lengths (blue line), and those obtained by stitching errors using two lengths placed end-to-end (black circles).

The results from the common path single-pass technique for the IFM and the ADM of the LT under test are shown in Figure 12a,b, respectively. The results from the common path double-pass technique are shown in Figure 12c,d. In each of the figures, we show the ranging errors from a single measurement, those obtained by stitching errors using three overlapping lengths, and those obtained by stitching errors using two lengths placed end-to-end.

Overall, the ranging errors obtained by stitching agree quite well with the errors obtained without stitching, except for the case shown in Figure 12b. The variation between the single measurement and the stitched measurement for the ADM in Figure 12b is likely due to changes in the temperature readings of the laser tracker under test even when the room itself is stable. This is also the likely cause of the small changes in slope between the single measurement and the stitched measurements in Figure 12a,c.

In the case of the IFM, stitching errors by overlapping three lengths produced nearly the same result as that obtained by stitching errors of adjoining lengths because the IFM has high repeatability; see Figure 12a,c. That is not the case for the ADM, where there is a small discrepancy between the two approaches.

The slope seen in the IFM error plots is due to the errors in the temperature and pressure readings of the LT under test. The slope seen in the ADM is somewhat larger than that for the IFM because of both errors in the temperature/pressure readings and uncorrected systematic errors in the ADM itself. 

### 4.5. MPE and Test Value Uncertainty

We discuss the MPE and test value uncertainty for the case of the common path double-pass approach because we can measure the full length of the LT under test. The discussion also applies to the common path single-pass approach. 

The MPE specification for the IFM of the LT under test is 0.5 µm/m. The MPE is, therefore, 1 µm at 2 m, 20 µm at 40 m, and 39 µm at 78 m. The MPE for a displacement of 38 m (from 2 m to 40 m) and 76 m (2 m to 78 m) are obtained by summing in quadrature, and thus, 20 µm and 39 µm, respectively. The observed range errors (shown in Figure 12c) are smaller than these specifications. The MPEs are shown in Table 2. The MPE specification for the ADM is 10 µm + 0.8 µm/m. The MPE is, therefore, 11.6 µm at 2 m, 42 µm at 40 m, and 72.4 µm at 78 m. The MPE for a displacement of 38 m (from 2 m to 40 m) and 76 m (2 m to 78 m) are 44 µm and 73 µm, respectively. The observed range errors (shown in Figure 12d) are smaller than these specifications. The MPEs are shown in Table 2.

The standard uncertainty for the reference interferometer measurements in our tape tunnel facility is 0.12 µm/m [10]. The standard uncertainty for a single 38 m and a single 76 m measurement are 4.6 µm and 9 µm, respectively. The expanded uncertainties (*k* = 2) are, therefore, 9 µm and 18 µm, respectively. These are shown in Table 2.

The uncertainty in the error for a 76 m length obtained by stitching errors of adjoining 38 m lengths could possibly also include the repeatability in the LT measurements at 38 m. Whether it is included depends on the definition of the test (the language in a documentary standard since we are discussing performance evaluation tests); see Section 6 for more on this. Assuming it is included and further assuming a standard deviation repeatability of 2 µm in the LT at 38 m, the standard uncertainty of the error in a stitched 76 m length is 9.2 µm. The expanded uncertainty (*k* = 2) is, therefore, 18.5 µm. These are shown in Table 2. The uncertainty in the error for a 76 m length obtained by stitching errors of overlapping lengths is slightly smaller than 9.2 µm but no smaller than that for a single measurement, which is 9 µm.

The measurement capability index (*C_m_*) is the ratio of the MPE to the expanded uncertainty. These are also shown in Table 2. As expected, it is not possible to achieve a *C_m_* larger than four for the IFM because the LT IFM and the reference interferometer have similar accuracies. We did, however, achieve a *C_m_* larger than four for the ADM because the MPE for the ADM was larger than that for the IFM. The process of stitching increases the test uncertainty by a small amount (because we chose to include the repeatability of the instrument under test in the test uncertainty) but does not affect the *C_m_* ratio significantly; thus, stitching is a viable option for range error evaluation of LTs. 

## 5. TLS Range Error Evaluation

We used an LT as the reference instrument for evaluating the range error of a TLS. Because the LT can provide 3D coordinate data, we did not only stitch errors but also reference lengths. We demonstrated the feasibility of stitching through two experiments. The first experiment was conducted in our temperature-controlled 60 m long tape tunnel facility, while the second experiment was conducted in a long indoor corridor between two buildings located underground where the temperature was only moderately controlled. In the next sub-sections, we describe the target used for the experiments, the measurements performed in the tape tunnel and long corridors, the results, and a discussion on test uncertainty and MPE. 

### 5.1. Target

**Figure 13:**
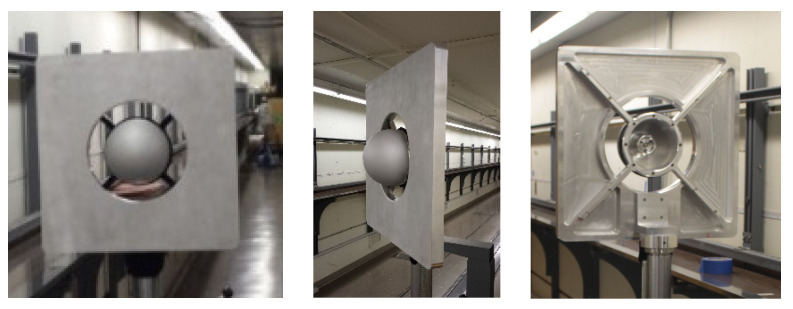
Plate-sphere target used for TLS range error evaluation with a 203.2 mm (8 in) mm diameter specialized sphere mounted centrally on a square plate with a side dimension of 609.6 mm (2 ft).

We use a plate-sphere target described in [48] for the experiments. The target consists of a large square media-blasted aluminum plate of a side dimension of 609.6 mm (2 ft) with a centrally located specialized sphere (also media-blasted aluminum) of diameter 203.2 mm (8 in), as shown in Figure 13. The sphere is hollow and contains a coincident three-point kinematic nest that allows an SMR to be seated. The sphere only serves as a fiducial, i.e., it helps locate a point on the plate that is common to both the LT and the TLS. The LT is placed at one end of the long measurement volume, while the TLS is placed at the other end. The plate-sphere target is mounted on a stand and faces the TLS. As it is moved from one position to the next, the TLS scans the plate while the LT records the coordinate of the SMR, thus allowing for the calculation of range errors. We obtained the ranging errors using both techniques—by stitching errors and stitching lengths. We next describe both approaches for the tape tunnel and the long corridor measurements.

In the case of a TLS, the target employed is large so that it can be seen from far distances. If a mirror must be placed close to position *A*, as in Figure 5c, the mirror must be large enough to be able to see the entire target. A mirror of such size and of high quality can be very expensive. Thus, using a mirror to fold the beam path of a TLS is not a viable option. We, therefore, did not perform stitching with the objective of using a smaller room to test the full range of the TLS. Rather, we performed stitching to exploit the high accuracy of an LT (which has a limited range) to test the full range of a TLS.

### 5.2. Measurements in the 60 m Tape Tunnel Facility

**Figure 14:**
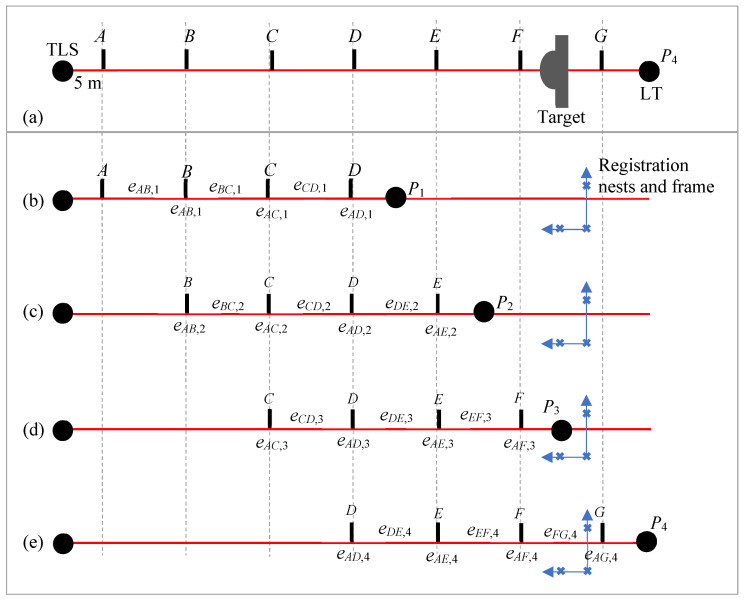
Stitching errors and lengths for TLS range error evaluation, (**a**) measuring the target at positions *AG* with the LT at position *P*_4_, (**b**) measuring the target at positions *A*–*D* with the LT at position *P*_1_, (**c**) measuring the target at positions *B*–*E* with the LT at position *P*_2_, (**d**) measuring the target at positions *C*–*F* with the LT at position *P*_3_, and (**e**) measuring the target at positions *D*–*G* with the LT at position *P*_4_.

We consider four positions (*P*_1_-*P*_4_) of the LT and seven positions (*A*–*G*) for the target, as shown in Figure 14. LT positions *P*_1_-*P*_4_ are nominally 10 m apart, and target positions *AG* are also nominally 10 m apart. Thus, the distance from *A* to *G* is 60 m. The TLS is about 5 m from position *A*. Our objective is to characterize the ranging errors of the TLS over a distance of 60 m. 

We first performed the range error measurement over the full 60 m distance to establish a reference against which we could compare the results obtained by stitching. For this purpose, the LT was located at *P*_4_. For each of the seven target positions *A*–*G*, the TLS scans the target to compute the center coordinate while the LT records the center of the SMR located inside the sphere. We then calculated the range error of positions *B*–*G* with respect to position *A*.

With the LT still at *P*_4_, we recorded the LT plate center coordinates and computed the TLS plate center coordinates (from a scan of the target) at the four target positions *D*–*G*. We also measured the coordinates of the registration nests located, as shown in Figure 14e. We then moved the LT to positions *P*_3_, *P*_2_, and *P*_1_ and repeated the measurements at the target positions shown in Figure 14b–d, respectively. At each position of the LT, we also measured the coordinates of the registration nests.

For each of the LT positions, the errors of each segment are shown above the corresponding segment in Figure 14. When calculating errors by stitching, the errors in these individual segments are used in the computation of the ranging error, as described in Section 3.1. When stitching lengths, i.e., performing stitching by registration, we can directly calculate errors with respect to position *A* for each LT position. These errors are shown at the target positions in Figure 14. These are used in the computation of the ranging error, as described in Section 3.2.

### 5.3. Measurements in a Long Corridor

The experimental setup is similar to that described in the previous section, except that LT positions *P*_1_–*P*_4_ are nominally 20 m apart, and target positions *A*–*G* are also nominally 20 m apart. Thus, the distance from *A* to *G* is 120 m. The TLS is about 5 m from *A*. Our objective is to stitch errors over 60 m in length to construct the errors over a 120 m length. Point spacing increases as the TLS moves farther from the target (angular spacing is constant), and at about 130 m from the TLS, there was barely any data acquired from the sphere to be able to compute the sphere center location. Thus, the size of the sphere on the target, not the length of the corridor, was the limiting factor in the maximum range we could measure.

### 5.4. Results

**Figure 15:**
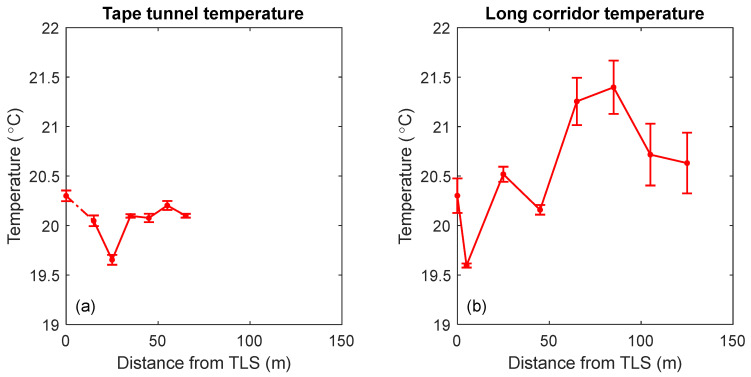
Average and standard deviation temperature over the duration of the experiment as a function of distance from the TLS (**a**) in the tape tunnel facility and (**b**) in the long corridor. The sensor placed 5 m from the TLS in the tape tunnel failed to record temperature; hence, the dashed line between the 0 m and the 15 m positions in (**a**).

Temperature is one of the largest contributors to the uncertainty in the reference measurements. We, therefore, recorded the temperature using eight loggers (~0.1 °C accuracy) during the measurement period. The loggers were placed near the TLS and at every target position. The temperature data are shown in Figure 15. The mean temperature in the tape tunnel was about 20.07 °C, one standard deviation at any target location for the duration of the experiment (~5 h) was about 0.05 °C, and the overall variation over the 60 m distance was ±0.3 °C. The mean temperature in the long corridor was about 20.57 °C, where the standard deviation at any target location for the duration of the experiment (~5 h) ranged from 0.02 °C to 0.3 °C, and the overall variation over the 120 m distance was ±1 °C. For the experiments, we set the temperature of the reference LT to 20 °C instead of reading the weather station of the LT because a single sensor is not sufficient to obtain a reasonable estimate of the average temperature along the beam path. Because the effect of temperature on the range is about 1 µm/m/°C, the maximum error in the range because of the spatial gradients in the long corridor was in the order of ±0.12 mm. We consider this error in the reference LT uncertainty.

Note that changes in air pressure over time also affect length measurements. The change in pressure over the duration of the experiment was about 2.5 mm of Hg. However, because the LT range values were corrected for pressure, the uncertainty in the range measurements because of pressure changes was small and could be ignored. Note that pressure changes along the beam path of the LT over 120 m is negligible; thus, spatial gradients were not as much of a concern for pressure as it was for temperature.

**Figure 16:**
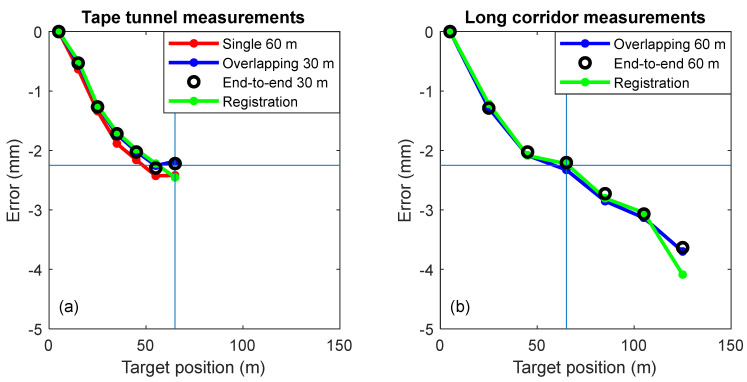
(**a**) Tape tunnel measurement-ranging errors from a single measurement (red line), which was obtained by stitching errors using four overlapping 30 m lengths (blue line), obtained by stitching errors using two 30 m lengths placed end-to-end (black circles), and obtained by stitching lengths (i.e., registration, green line). (**b**) Long corridor measurement-ranging errors obtained by stitching errors using four overlapping 60 m lengths (blue line), which were obtained by stitching errors using two 60 m lengths placed end-to-end (black circles), and obtained by stitching lengths (i.e., registration, green line).

Figure 16a shows the relative range of errors obtained in the tape tunnel over a 60 m range (i.e., from 5 m to 65 m with respect to the TLS) for four means—a single 60 m measurement, stitching errors of four overlapping 30 m lengths, stitching errors of two 30 m lengths placed end-to-end, and stitching lengths by registration. We observed excellent agreement between the methods. Figure 16b shows the relative range errors obtained in the long corridor over a 120 m range (i.e., from 5 m to 125 m with respect to the TLS) through three means—stitching errors of four overlapping 60 m lengths, stitching errors of two 60 m lengths placed end-to-end, and stitching lengths by registration. Again, there is excellent agreement between the methods. The blue crosshair in the figures highlights the agreement in the error at the 65 m position, which is about −2.25 mm, obtained both in the tape tunnel as well as in the long corridor.

### 5.5. MPE and Test Value Uncertainty

The standard deviation ranging accuracy specification for the TLS under test was 1.2 mm + 10 µm/m. The MPE specification based on three times the standard deviation was 3.75 mm at a distance of 5 m, 5.55 mm at 65 m, and 7.35 mm at 125 m. The MPE specification for a displacement of 60 m (from 5 m to 65 m) and 120 m (from 5 m to 125 m), were, 6.7 mm and 8.25 mm, respectively. The observed range errors (shown in Figure 16) were smaller than these specifications. The MPEs are shown in Table 3.

Next, we calculated the uncertainty in the reference measurements made by the LT. We only showed these calculations for the long corridor because the spatial temperature gradients in that corridor were significantly larger than in the tape tunnel; therefore, the uncertainty in the LT measurements was larger. If there are no spatial temperature gradients, the MPE specification for the ADM of the LT used as a reference was given by 10 µm + 0.8 µm/m. However, we knew that there were spatial gradients of the order of ± 1 °C in that corridor. Because the effect of temperature on range is about 1 µm/m/°C, the specification for the ADM was 10 µm + 1.8 µm/m. The MPE for a displacement of *d* m, i.e., from 5 m to *d* + 5 m, was then given by $\sqrt{{\left(10+1.8\times 5\right)}^{2}+{\left(10+1.8(d+5)\right)}^{2}}$. Assuming a uniform distribution and any value within this bound as equally likely, the standard uncertainty was $1/\sqrt{3}$ times the MPE. Thus, for a displacement of 60 m (from 65 m to 5 m—note that when the target was about 5 m from the TLS, the LT was about 65 m from the target), the MPE was 127 µm, and the standard uncertainty was 73 µm. The expanded uncertainty was, therefore, 0.147 mm. The measurement performance index ratio, *C_m_*, is 46 (6.7 mm/0.147 mm), which is substantially larger than the typically required value of four, indicates that the LT is suitable as a reference instrument to evaluate the TLS. Note that this is for a single measurement, i.e., not for the stitching case.

When stitching errors of two adjoining 60 m lengths are used to construct the errors of a 120 m length, the uncertainty in the LT measurement doubles; thus, the standard uncertainty due to the LT and temperature gradients is 0.147 mm. As mentioned in Section 4.5 for the case of stitching adjoining segments, it is possible that the repeatability of TLS might have to be included in the test uncertainty. Whether it is included depends on the definition of the measurand (the language in a documentary standard if it is a performance evaluation test); see Section 6 for more on this.

Assuming this is included and further assuming that the standard deviation repeatability is on the order of 0.1 mm at 60 m, the standard uncertainty is 0.178 mm, and the expanded uncertainty (*k* = 2) is 0.356 mm. The measurement performance index, *C_m_*, is 23, which is substantially larger than the typically required value of four, indicating that stitching errors using an LT as a reference instrument is a viable solution to evaluate the TLS. Stitching errors of overlapping lengths reduces the uncertainty by a small amount. 

When stitching reference lengths by registration, the uncertainty in the registration process must be included along with uncertainty due to the LT and temperature gradients. Again, see Section 6 for more on this. We estimate the uncertainty in the LT measurements due to registration to be about 0.05 mm based on the location of the registration nests with respect to the LT and using ADM MPEs. Thus, the standard uncertainty in the LT measurements is 0.178 mm. The expanded uncertainty (*k* = 2) is, therefore, 0.356 mm. Again, *C_m_* is substantially larger than the typically required value of four, indicating that stitching lengths using an LT as a reference instrument is a viable solution to evaluate the TLS.

## 6. Discussion on Test Uncertainty

In this section, we address the question of whether the repeatability of the instrument under test should be included in the calculation of the test uncertainty when stitching errors. The test protocol (i.e., the detailed specification of a test codified in the documentary standard) defines the test measurand. In many cases, the measurand follows from the words in the test in a straightforward and unambiguous manner. Because the measurand is unambiguous, the evaluation of the test value uncertainty can also follow in a straightforward manner. However, ambiguities may arise from variations in the precise words chosen within the documentary standard. These variations can lead to different interpretations of the measurand and, consequently, different components of uncertainty being considered in the evaluation.

Suppose we are interested in evaluating the ranging error of an IUT over 60 m. The primary method involves calibrating and measuring a 60 m length directly with the RI. Because we either do not have a room large enough to perform this test or an RI with a range of 60 m, we consider the case of stitching errors in adjoining 30 m lengths to evaluate the error over 60 m. In that case, the alternative method is the measurement of a 30 m length twice under specific conditions given for each, with the results combined to assess the IUT’s performance. The evaluation of the test value uncertainty depends on how the test protocol handles the combination.

Case 1: The two (signed) observed errors (with each error being the indicated value of length minus the calibrated value) are added. In this case, the test protocol indicates that if the sum of the errors is bounded by +/− MPE (or, more correctly, if the sum of the errors yields a pass according to the decision rule), then this test passed. Here, there is no reason for the repeatability of the instrument under test to be included in the test value uncertainty evaluation.

Case 2: The two measured lengths are used to answer the following question: given the information gained from the two 30 m measured lengths, can we predict what the measured length would have been had the instrument measured a single 60 m length? This prediction, minus two times the calibrated value, is then compared with the MPE to see if this test passes. In this case, the best prediction that can be made about the length the instrument would have measured in the 60 m case would be the sum of the two 30 m lengths. However, there would be uncertainty associated with the prediction that arises from the repeatability of the instrument.

In many cases, the actual wording of a standard does not use the exact words used in the cases above. One must decipher which case applies from the words given. For example, the test protocol might not use the words “predict” as used above, but rather a word like stitched might be used maybe without clarification, indicating which of the two cases shown above applies.

When we stitch two short lengths by registration to construct a long reference length, the definition of the test is the error in the overall long length; therefore, the uncertainty from the registration process must be included in the calculation of the test uncertainty. 

Finally, we note that in the case of TLSs, we have a choice as to whether to stitch errors or to stitch lengths. If the repeatability of the TLS is small compared to the errors introduced by registering the LT, it is advantageous to stitch errors rather than stitch lengths. If the repeatability of the TLS is large in comparison to registration errors of the LT, it is advantageous to stitch lengths by registration. 

## 7. Conclusions

Evaluating the range of errors of LTs and TLSs is a challenging problem. Many users of LTs do not have rooms large enough to evaluate the full range of their instruments, while users of TLSs sometimes do not have access to a reference instrument whose uncertainties are smaller than the accuracy specification of their TLSs. We propose the use of stitching as a means to overcome these challenges. 

In the case of LTs, we proposed stitching errors of adjoining or overlapping lengths to obtain the errors over the full range of the LT in a room that is only about half or one-fourth the maximum testing range of the LT. We demonstrated the validity of this technique in our temperature-controlled laboratory by comparing it against a single long-length measurement. 

In the case of TLSs, we proposed two methods of stitching—the first by stitching errors similar to LTs and the second by stitching lengths, i.e., registration, where we transform reference LT data to a common frame through the use of registration nests. Stitching allowed us to use an LT as a reference instrument and realize uncertainties in the reference measurements that are substantially smaller than the accuracy specification of the TLS. We demonstrated the validity of these techniques in both our temperature-controlled laboratory and in a moderately temperature-controlled long corridor. 

Stitching errors and stitching lengths can possibly increase the test uncertainty by a small amount in comparison to a single un-stitched measurement. However, the measurement capability index does not change appreciably, indicating that stitching is a viable solution for range error evaluation of LTs and TLSs.

## Figures and Tables

**Table 1:** Interferometric benches offered by NMIs and expanded uncertainty in reference lengths.

Country	Expanded Uncertainty (*k* = 2)	Description
United States	0.24 µm/m	NIST has a 60 m long interferometric bench [6,8,10] with a rail and carriage that is used for measuring tapes, fiber optic cables, and the ranging units of LTs.
Poland	5 µm + 0.5 µm/m	The Central Office of Measures, Poland, maintains a 50 m bench [11] for the calibration of steel tapes, electronic distance meters (EDMs), laser interferometers, and LTs.
China	0.1 µm + 0.1 µm/m	The National Institute of Metrology, China, has established an 80 m long granite guideway-based system [12] for the measurement of total stations, LTs, TLSs, laser range finders, etc.
Finland	$2\sqrt{{\left(0.24\right)}^{2}+{\left(0.043L\right)}^{2}}$ µm, *L* is in meters	The Center for Metrology and Accreditation, Finland (MIKES) has a 30 m bench [13] for this purpose.
Italy	0.6 µm/m.	The Istituto Nazionale di Ricerca Metrologica (INRiM) in Italy has established a 28 m bench [14].
Japan	50 µm + 0.4 µm/m	The National Metrology Institute of Japan (NMIJ) has a 100 m optical bench for EDM calibration [15].

**Table 2:** MPE, expanded uncertainty *U* (*k* = 2), and measurement capability index *C_m_*.

	MPE_IFM_ (µm)	MPE_ADM_ (µm)	*U* (µm)	*C_m_*, *_IFM_*	*C_m_*_, *ADM*_
Single 38 m	20	44	9	2.2	4.7
Single 76 m	39	73	18	2.2	4.0
Stitching errors 76 m ^1^	39	73	18.5	2.1	3.9

^1^ See Section 6 for more on whether the repeatability of the LT must be included in the test uncertainty when stitching errors of adjoining segments.

**Table 3:** MPE expanded uncertainty *U* (*k* = 2), and measurement capability index *C_m_*.

	MPE	*U* (*k* = 2)	*C_m_* (=MPE/*U*)
Single 60 m	6.7 mm	0.147 mm	46
Stitching errors 120 m ^1^	8.25 mm	0.356 mm	23
Stitching lengths 120 m ^2^	8.25 mm	0.311 mm	27

^1^ See Section 6 for more on whether the repeatability of the TLS must be included in the test uncertainty when stitching errors of adjoining segments. ^2^ The uncertainty in the registration process is included in the calculation of the test uncertainty.

## Data Availability

Data is contained within the article.
